# Prudence and Gutta-Percha in Crown- and Bridge-Work

**Published:** 1893-10

**Authors:** George V. I. Brown

**Affiliations:** Duluth, Minn.


### PRUDENCE AND GUTTA-PERCHA IN CROWN- AND BRIDGE-WORK.[1]

BY GEORGE V. I. BROWN, DULUTH, MINN.

My desire is to avoid, so far as possible, the detail of mechanical construction, and deal with the broad underlying principles that govern the attachment of a foreign substance to a root in the living mouth, as a substitute for nature's perfect crown-work, and the yoking of one tooth to another to support substitutes, and calling upon them to perform duties belonging to the full number.

Experience has certainly given this class of work an unquestioned right to be considered among the useful and therefore reputable operations in the oral cavity; and the query naturally arises, "What has been the effect of this introduction upon the general practice of dentistry?" and a glance at the record seems to prove that the result has been a marked one both for good and for ill.

The benefits have been the preservation and restoration to a state of usefulness of many roots and broken-down crowns of teeth that would otherwise have been lost, thus putting off the evil day when artificial dentures would be necessary.

Again, the result of this effort to utilize such roots has more than anything else influenced the wonderful advance in the therapeutic

[1] Read before the World's Columbian Dental Congress, Chicago, August, 1893.

methods by which they are restored to healthful usefulness, and diseased conditions removed.

An advance has been made, in that while the average dentist was almost totally ignorant of metal plate-work, to-day every student is required to have sufficient skill in this direction to be able at least to make an artistic-looking gold crown. On the other hand, instead of acquiring greater perfection in filling, the temptation has many times been too great to cover teeth with large cavities but good remaining structures in this manner, until the wholesale destruction of natural crowns that might properly have been filled is something to look upon, and unless the tendency be checked yet more terrible to contemplate in its future aspect.

Many times, too, there has been a total loss of good roots and even whole teeth through injudicious attachment for the purpose of sustaining a strain they were unable to bear.

It is a well-known principle that a thing can be no stronger than its weakest part. This in crown-work would mean that the following points particularly need protection, which the perfect crown must provide:

*First.* Attachment to secure without fear of dislodgement.

*Second.* A natural appearance if for the anterior teeth.

*Third.* Provision against the action of destructive agents at or near the gingival line.

*Fourth.* Secured against danger of splitting by a band, or its equivalent, by forming the joining surfaces of root and crown in such manner as to equalize the strain.

*Fifth.* The imitation of the natural form of the crown upon approximal and occluding surfaces.

Bridge-work embodies dangers, increased proportionately to the greater strain it is necessarily called upon to bear and its difficulty of adjustment, with the added risks of the perhaps doubtful capability of a few roots to perform the natural service of several, besides having to support the sometimes not inconsiderable weight of the attachment, and must be so formed that the collection of particles of food and other readily decomposed matter between the bridge and the membrane covering the ridge under it may be avoided by making the surface self-cleansing so far as possible.

Having enumerated the requirements, an examination of the various methods seems to be in order, and I begin with the broad-sounding but quite true statement that crowns inserted with any of the oxychloride or oxyphosphate cements, without band or other protection at the joining with the root, no matter how secure the pin may be, must be considered as of a temporary nature, for no matter how perfectly the surfaces may be adjusted, disintegration will surely follow, and not only cause loosening of the crown but allow more or less destructive action upon the root itself.

The use of amalgam as a setting is, of course, a great improvement upon cement in crowns of this class, but even amalgam is not always proof against bacterial influences at that critical point, the gingival line, as shown by the frequent failure of fillings. The force of the jaws applied in general use, or an accidental blow, not infrequently causes that most serious of possible troubles, the splitting of the root perhaps beyond repair, unless these crowns are protected with a band, a thing not ordinarily practicable in using amalgam, or by making the end of the root convex, and having a concavity in the crown to fit over it, by grinding slanting surfaces externally and internally from a point in the centre, giving a "V" form, or taking some other precaution to equalize the strain so far as possible, and distribute its effects all around the root instead of being applied to one side without the assistance of the support of the other.

The Logan, Bonwill, Howe, and other *all*-porcelain crowns most commonly used with cement and amalgam, as described, have certainly the advantage to some extent in natural appearance, which must be conceded for the six anterior teeth over other methods which require the use of a plate-tooth with backing.

Noting the value of a band for the security of the crown, a special consideration of its application is next demanded.

The perfectly-fitting band, as described by the facile pens of writers and the positive statements of discussers of the subject, to the experienced mind, must be much modified to conform to the hard facts of daily practice. Granted that upon a single reasonably sound root, ground down and stripped of its enamel, with the gum in a normal healthy condition, a band can be so fitted that when set with cement there will be but little if any fear of disintegrating action of the secretions; grant still further that this would also hold good where two such roots were situated in the mouth in suitable position, so nearly straight in the jaw as to admit of the completed piece being passed over each at the same time and in the same direction, then ask ourselves how many of all the broken-down and once diseased roots that we desire to save would correspond to that description, and of all the bridge cases we have and are caring for, in how many are the roots so conveniently situated; add to these the consideration that in all other cases more or less opportunity must be offered for the secretions to act upon the cement, knowing as we do that it offers but very uncertain resistance to such action, and we realize at once this weak point in present methods, and the need of some improvement in this direction.

The teachings of Dr. Flagg and his followers, while, perhaps, going further than could be generally recommended, have at least been instrumental in establishing the value of gutta-percha in its capability to withstand the action of the acids of the mouth, and as a preservative against caries even where every other filling-material would fail; its only disadvantage being that it wears away too readily in exposed positions; but while its good properties are of the first importance in crown-work, the softness does no harm, because it is protected from wear and will not disintegrate. The advantage may be summed up before proceeding to more detailed description under the following heads:

*First.* It is impervious to acid secretions or bacterial influences.

*Second.* Not irritating to surrounding tissues.

*Third.* Easily removed when necessary.

As against these three there is simply one consideration, viz., the difficulty of adjustment. The value of these properties can best be understood by contrasting with other methods generally advocated.

The system once so positively advanced, but in the light of civilized progress apparently losing advocates, which required the snapping off of natural tooth-crowns, the destruction of pulps by driving a wooden point into the living contents of freshly uncovered canals, and the withdrawal of the pulp with the wood, is to my mind unprofessional to a degree bordering closely upon the barbaric.

The most elaborate piece of amalgam-work that I have ever seen, having been under my observation for some months past, has convinced me that first of all such manipulation in average hands would be out of the question; and while I doff my hat in deference to the wonderful ingenuity and great skill which enabled the well-known operator to build solid masses of amalgam from one loose tooth to another, and even across the vacant spaces where teeth had been lost in both jaws, anchoring it firmly, and building up most shapely dummy crowns of this material, it did seem to be a most unnecessary expenditure of talent in view of simpler and much safer methods.

Dr. E. Parmly Brown has undoubtedly done much with his system of porcelain bridges anchored with gold fillings, malleted about a bar extending into the tooth, and here again is an example of what individual skill can do; but from the stand-point of the less skilful average practitioner, there are some points which seem to require criticism in comparison with other needs.

For firmly fixing the position of teeth loosened by pyorrhœa, bands carefully fitted, soldered together, and cemented in place are a step in advance, but they also allow the pressure upon the crowns to work to their disadvantage, and the cement gives out so soon that they can hardly be considered of any paramount value.

Filling with gold about a gold wire embedded in the cutting-edges of lower incisors is not only an exceedingly laborious, but it is also a difficult and uncertain operation to impact the gold securely when the teeth are not held so firmly that movement is next to impossible; and it is almost an impossibility to do it, because not only are such teeth capable of forward and backward, lateral and circular movement, but upward and downward as well, andthus making a very poor resistance to the necessary force in condensing the gold, without which the operation is valueless.

The anchoring of amalgam upon each side and building across from one to the other, even in most skilful hands, seems to be insufficient except in very favorable cases, besides being, as already stated, most unsightly. But by dressing the sides of the tooth as straight as possible up and down, grinding enough of the cutting-edge to allow of a covering of gold without interfering with the occlusion, and bevelling to let the enamel-edge hide so far as possible the gold covering, a metal die made from a cast and perfect impression, the crown of gold plate struck up and made to accurately fit the cast, and the front part afterwards cut out, these all put in place in the mouth, an impression taken and all soldered together, then set with gutta-percha, the result is that each tooth not only is firmly held in position with its neighbor, but the edges are so covered that no upward and downward movement can occur, and we know that gutta-percha, while it may wear off a little at the exposed edges, will not disintegrate under the gold covering, therefore that operation may be regarded as a permanent one, besides being less unsightly than most of the other methods, which do not require cutting off the tooth.

This method applied to the abutments for bridges where any of the anterior teeth remain in a condition sufficiently sound to warrant preservation of the natural crown, avoids the destruction of nature's handiwork as shown in a living, nourishing, healthful pulp and natural tooth-structure, gives an attachment that is absolutely secure and proof against every force that may be applied without fear of checking porcelain fronts, can be carried up beyond the gingival margin, left a little short of it, or taken to the enamel line just as the surrounding conditions seem best to warrant, because of the trustworthiness of gutta-percha under fire of the secretions.

To one accustomed to use cement for this purpose the difficulty of applying gutta-percha as a substitute seems almost insurmountable; but a moment's recollection of the trials attendant upon the setting of his first crowns will recall the fact that cement too had its difficulties for the beginner.

For gold crowns, where teeth are covered entirely, I prefer the red gutta-percha we get in form of sheets. It has a degree of toughness that is valuable, but for anterior crowns with open faces the red line at the edge of the openings is undesirable, and I therefore use some white preparation that softens at a low temperature, as this property greatly facilitates its handling in the mouth. There being no danger to apprehend from wear, hardness is not so desirable as for filling.

It is my custom to heat both crowns and setting-material upon a tray to protect from the open flame, spread with warm instruments a thin coating over the inner surface of the crown, which should be first roughened on the inside by scratching with an instrument, aiming to have as nearly as possible the exact amount necessary, but depending upon a vent to get rid of a small amount of surplus; then after drying the surface of the teeth with alcohol and warm-air blast, wipe with some suitable germicide, saturate a pledget of cotton in eucalyptus, coat the surface with it to prevent the too rapid drying of the chloro-percha, of which a thick solution is then applied around the neck, so that when the crowns already heated as hot as they can be handled in the fingers and prepared as described are driven home, the eucalyptus, chloro-percha, and gutta-percha in the crowns will form a thick creamy mass that will penetrate and perfectly fill every space about the necks, and, even if surplus be not removed afterwards, will not cause irritation as might a particle of cement in the same position.

Sometimes too much gutta-percha will prevent the crown going quite into the proper position, just as sometimes happens with cement; but the remedy is much simpler, because easily removed while still warm; or if, for accidental or other causes, removal be desired afterwards, hot water or hot air applied will in a short time heat the metal sufficiently to soften the material and facilitate the separation without the necessity of damaging the crown, and any one who has tried to pull them off without first warming will be quite satisfied that any properly-adjusted crown can be securely held in this manner.

